# Exploring the role of gut probiotic metabolites in the prevention and treatment of otitis media

**DOI:** 10.3389/fcimb.2025.1661871

**Published:** 2025-08-15

**Authors:** Bo Zheng, Haiyong Jin

**Affiliations:** Department of Otolaryngology, The Second Affiliated Hospital & Yuying Children’s Hospital of Wenzhou Medical University, Wenzhou, Zhejiang, China

**Keywords:** otitis media, probiotics, metabolites, network pharmacology, gut microbiota

## Abstract

**Introduction:**

The gut microbiota derived metabolites show significant therapeutic effects on otitis media, yet the specific active metabolites and mechanisms involved remain undocumented. The primary objective of the study was to utilise a network pharmacology approach to investigate the active metabolites and underlying mechanisms by which gut microbiota exerts their effects against otitis media.

**Methods:**

A set of 110 gut microbiota-derived metabolites was retrieved from the MiMeDB database. Their target genes were identified using SEA (Similarity Ensemble Approach), resulting in 6860 human target genes. Parallelly, a differential expression analysis using the GEO dataset identified dysregulated genes in otitis media. Upon intersecting these with the metabolite target genes, we identified 268 common genes, which likely represent molecular mediators through which microbial metabolites exert its effects in otitis media. PPI interaction was used to identify the 10 hub targets. To understand the post-transcriptional regulation of these common genes, we identified miRNAs targeting them using the multiMiRR package.

**Results:**

The functional enrichment and disease association analyses of these genes and miRNAs revealed their significant involvement in inflammatory and immune regulatory pathways, many of which are shared with chronic otitis media pathogenesis.

**Discussion:**

Overall, this integrative approach established a strong link between gut microbial metabolites, their host gene targets, and miRNA-mediated regulatory mechanisms in otitis media. This study provided comprehensive insights warranting additional research on the therapeutic potential of metabolites for otitis media.

## 1 Introduction

Otitis media, commonly referred to as middle ear infection, is a group of inflammatory conditions that affect the middle ear and are frequently caused by infections from viruses or bacteria (Singh et al., 2021). Otitis media includes acute otitis media (AOM), which is a sudden and painful infection, otitis media with effusion (OME), also known as “glue ear”, where fluid accumulates without active infection, and a long-term condition characterized by ongoing ear discharge due to a perforated eardrum known as chronic suppurative otitis media (CSOM). These disorders are characterized by fluid buildup and inflammation behind the eardrum, which can cause pain, fever and temporary hearing loss in patients. While it is more common in children, it can also affect people of all ages (Schilder et al., 2016) *Streptococcus pneumoniae*, *Moraxella catarrhalis* and *Haemophilus influenzae* are some of the primary disease causative agents (Zahid et al., 2024). In most cases, AOM resolves without antibiotic treatment, however antibiotics are prescribed when the infection is severe or recurrent to prevent complications. At the same time, these treatments are limited to reducing antibiotic resistance development to some extent (Schilder et al., 2017). Pneumococcal and influenza vaccinations are examples of preventive measures that have decreased the incidence of disease, yet, they do not cover all pathogenic organisms, underscoring the need for more vaccine development (Zahid et al., 2024). Despite the advancements, otitis media remains a major reason for the surgeries, antibiotic prescriptions and hospital visits among children. This underscores the need for more research and development in the field of diagnosis, prevention and treatment of otitis media.

Recent studies propose that there is a complex relationship between otitis media and gut microbiome, especially in children. It is proven that there is only a limited difference between the gut microbiota composition between patients with recurrent AOM and normal subjects, though certain bacterial taxa, such as Turicibacter, showed associations with the frequency of infections, indicating a possible link which needs further exploration (Florjan et al., 2024). As mentioned earlier, the antibiotic treatment for AOM can disrupt the gut microbiome, which may increase the risk of colonization by resistant microbial species, especially in children lacking beneficial bacteria like Blautia, Ruminococcus, and Faecalibacterium (Li et al., 2022). Recent studies increasingly suggest the gut microbiome modulation using probiotics as a therapeutic intervention in treating diseases like otitis media. Some evidence also says that combining probiotics with reduced antibiotic use may improve the clinical outcome and reduce inflammation (Godur et al., 2023). Additionally, research also highlights the protective role of upper respiratory tract bacteria such as lactic acid bacteria and Streptococcus salivarius, which may inhibit OM pathogens and could be developed as targeted probiotics (Jörissen et al., 2021). However, the field is still evolving, and more longitudinal and standardized studies are needed to clarify the gut–ear axis and to develop effective microbiome-based interventions for OM (Florjan et al., 2024; Godur et al., 2023; Tamir et al., 2023).

Probiotic metabolites, also called postbiotics, are the bioactive compounds produced by probiotic species that can play a significant role in the prevention of disease and treatment. Most significant examples of probiotic derived metabolites are short-chain fatty acids (SCFAs), bacteriocins, and exopolysaccharides, they are known to exert anti- inflammatory effects, immune response modulation, and pathogenic bacteria growth inhibition, thereby improving treatment outcomes in various diseases including inflammatory bowel disease (IBD), cancer, depression, and periodontitis (Deandra et al., 2023; Petrariu et al., 2024).

We hypothesise that gut microbial metabolites derived from probiotics possess therapeutic potential in the treatment of otitis media through modulating host gene and miRNA expression involved in key inflammatory and immune response pathways. Given the current limited evidence available regarding the specific role of probiotics derived metabolites in the context of otitis media, we employed a bioinformatics approach to find out how these metabolites might work as therapeutic agents. The ability of microbial metabolites to alter genes and miRNAs implicated in important inflammatory and immune response pathways linked to otitis media was revealed by our analysis, which underscores their potential as new therapeutic targets.

## 2 Materials and methods

### 2.1 Collection of probiotic gut microbiota metabolites

Firstly, we collected metabolites of probiotic species present in the gut, specifically *Lactobacillus acidophilus, Lactobacillus fermentum*, *Bifidobacterium bifidum*, *Bifidobacterium lactis*, and *Streptococcus thermophilus* species from the MiMeDB database (https://mimedb.org/) (accessed on 21 June 2025).

### 2.2 Identification of the targets of gut microbiota-derived metabolites

The PubChem database was used to retrieve the canonical SMILES of each metabolite (https://pubchem.ncbi.nlm.nih.gov/). Then, the genes targeted by the metabolites were retrieved using the similarity ensemble approach (SEA) (http://sea.bkslab.org/), which we accessed on 22 June 2025.

### 2.3 Identification of differentially expressed genes in otitis media

In this study, dataset GSE49128, obtained from Gene Expression Omnibus, was evaluated to identify otitis media associated differentially expressed genes (DEGs), Differential expression analysis was performed using the GEO2R web tool, which utilizes the limma package in R. Quantile normalization was applied to standardize expression levels across samples. DEGs were identified based on adjusted p-value < 0.05 (Benjamini-Hochberg correction) and |log2 fold change| ≥ 0.5 as thresholds. The dataset included the gene expression profile from a mouse model, comprising of nine samples) treated with trans-tympanic heat-killed *Hemophilus influenza* and untreated eight untreated control samples. Statistically significant DEGs were found based on adjusted p-values (Suganya et al., 2022). Then, the DEGs identified from the mouse model of otitis media were annotated using their corresponding human orthologs to facilitate downstream human-specific analyses.

### 2.4 Collection of gut microbe targets against otitis media

Gut microbe-based therapeutic targets were identified by overlapping the DEGs and the genes targeted by gut microbial metabolites. Further, the identified target genes were used for the subsequent computational analysis (Saranya and Viswanathan, 2024).

### 2.5 Protein-protein interaction network analysis

The Protein-protein interaction (PPI) network was constructed using STRING analysis (https://string-db.org/) to find specific interactions and functional associations among genes and proteins linked to otitis media (Sudha et al., 2022). After obtaining PPI interaction from the string, the final output was visualised using Cytoscape 3.10.3. Then, the top 10 targets were identified using the CytoHubba plugin to find the key molecular interactions. The top targets having the highest degree values in the PPI networks were considered a hub target to control the interaction network associated with otitis media.

### 2.6 GeneMania analysis

The biological network associated with the target genes was explored using GeneMania (http://www.genemania.org/), which gives information on protein and genetic interaction pathways, co-expression, co-localization, and protein domain homology (Aswathy et al., 2024).

### 2.7 Gene ontology and pathway enrichment analysis

To gain insights into the biological and molecular mechanisms of hub genes, enrichment analysis was carried out. For this, gene ontology was performed using ShinyGo 0.82, which consisted of “molecular function (MF), biological function (BF), and cellular component (CC)” analysis (Ge et al., 2020).

### 2.8 miRNA–gene interaction network

We used multiMiR, which is an R package to retrieve the miRNAs that target hub genes and predicted miRNA–gene interactions from multiple databases, including miRTarBase, TargetScan, and miRDB (Holý et al., 2023).

### 2.9 KEGG pathway analysis

DIANA-miRPath v3.0. was used to analyse KEGG pathway enrichment of the identified miRNAs to reveal the potential signaling pathways associated with identified miRNAs against otitis media (Tastsoglou et al., 2023).

### 2.10 miRNA-disease association prediction

miRNet was utilised to predict gene-disease associations to identify miRNAs which influence biological processes associated with various diseases, based on KEGG database. The degree association score for each miRNA was calculated based on network pharmacology, where nodes represent hub targets and diseases. Whereas, edges denote the strength of interaction with the diseases. A strong association with the disease was indicated by the higher number of interactions (Chang et al., 2020).

## 3 Results

### 3.1 Acquisition of metabolites derived from probiotic gut microbiota

We collected the metabolites derived from *Lactobacillus acidophilus, Lactobacillus fermentum*, Bifidobacterium bifidum, *Bifidobacterium lactis*, and *Streptococcus thermophilus* species using the MiMeDB database. A total of 110 human gut microbiome derived metabolites were identified as significant to study the therapeutic effects of gut microbiota. The list of identified metabolites is provided in **Supplementary Table 1**.

### 3.2 Identification of molecular targets of gut microbiota-derived metabolites

Molecular targets of identified metabolites were predicted using Similarity Ensemble Approach (SEA) based on their SMILES structure, resulting in the identification of 6860 target genes (**Supplementary Table 2**).

### 3.3 Collection of potential targets for otitis media

**Figure 1:**
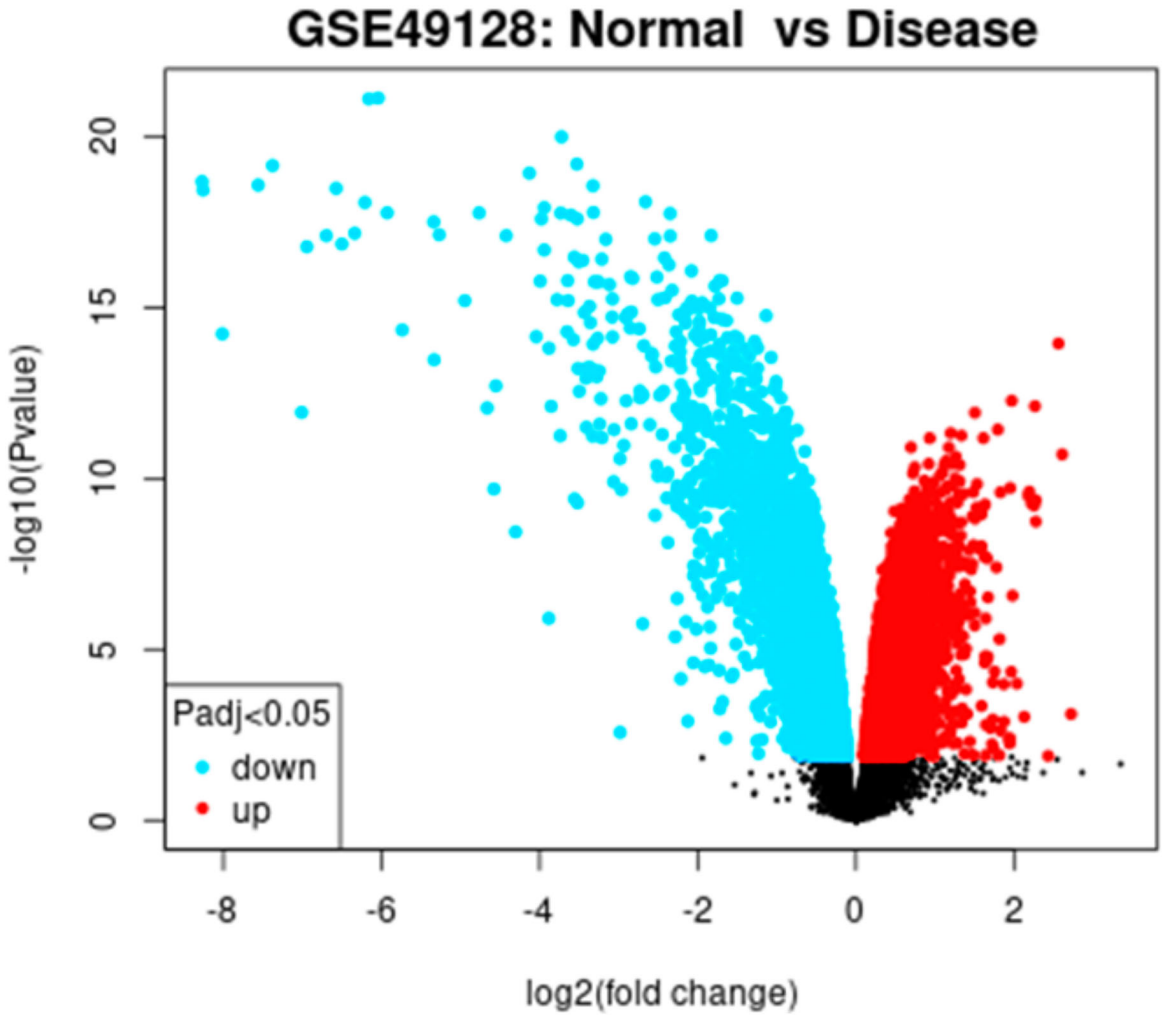
Volcano plot illustrating differentially expressed genes (DEGs) between otitis media samples and controls. Red dots indicate significantly upregulated or downregulated genes, while black dots represent genes with no significant change. The vertical dashed lines denote the threshold cutoffs for statistical significance.

To identify the targets specific to otitis media, we performed differential gene expression using the GSE dataset. Based on the selection criteria of an adjusted P-value < 0.05 and absolute log fold-changes ≥ 0.5, about 12812 genes were identified as significant DEGs. The distribution of DEGs is illustrated by a volcano plot presented in **Figure 1**.

### 3.4 Collection of gut microbe targets against otitis media

**Figure 2:**
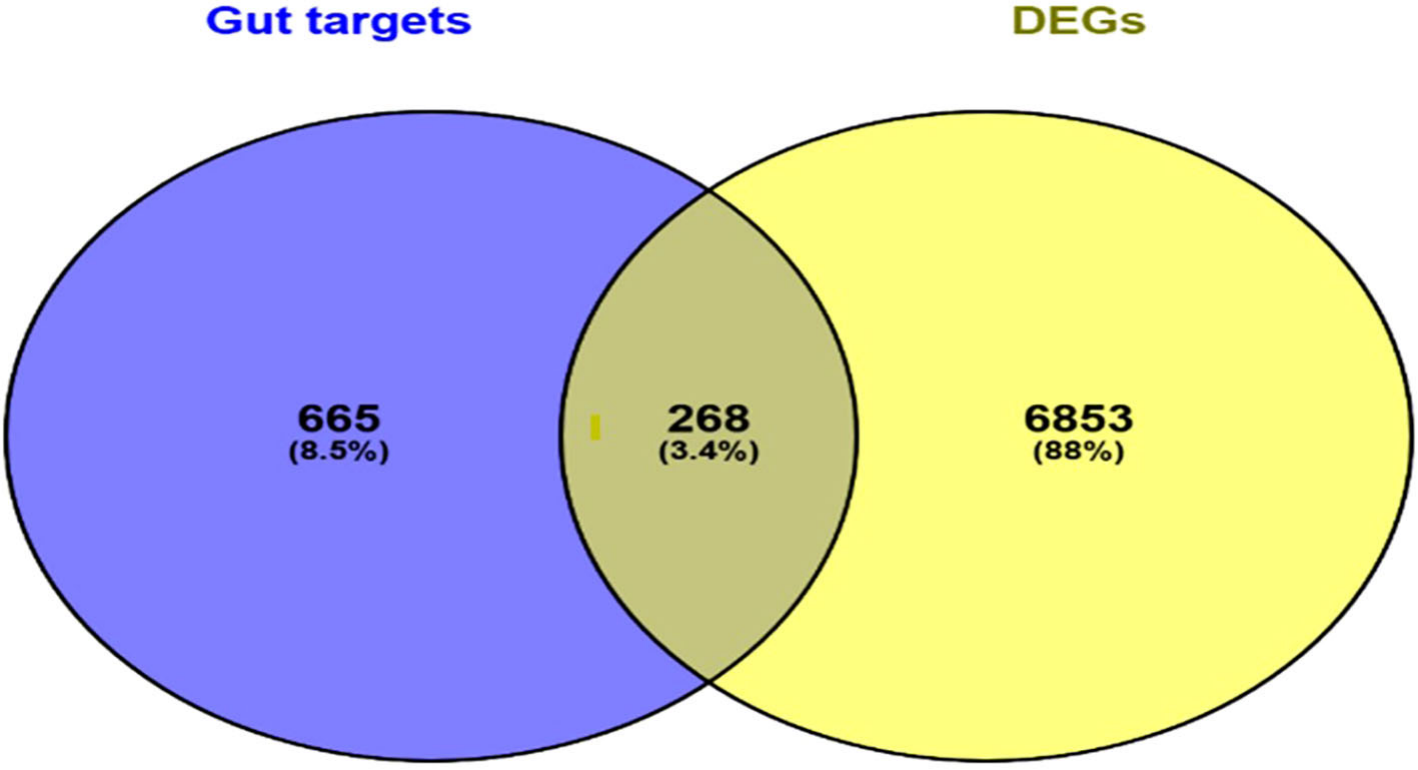
Venn diagram depicting the overlap between predicted gut microbial metabolite target genes and DEGs identified in otitis media. The common genes represent potential microbial targets associated with otitis media pathology.

A total of 268 genes were found common between both the DEGs and molecular targets of gut microbiota-derived metabolites (**Figure 2**) (**Supplementary Table 3**). Those genes were considered as the potential gut microbial targets against otitis media, implying that probiotic metabolites could modulate the same pathway or genes that are dysregulated in otitis media.

### 3.5 PPI network of gene targets

**Figure 3:**
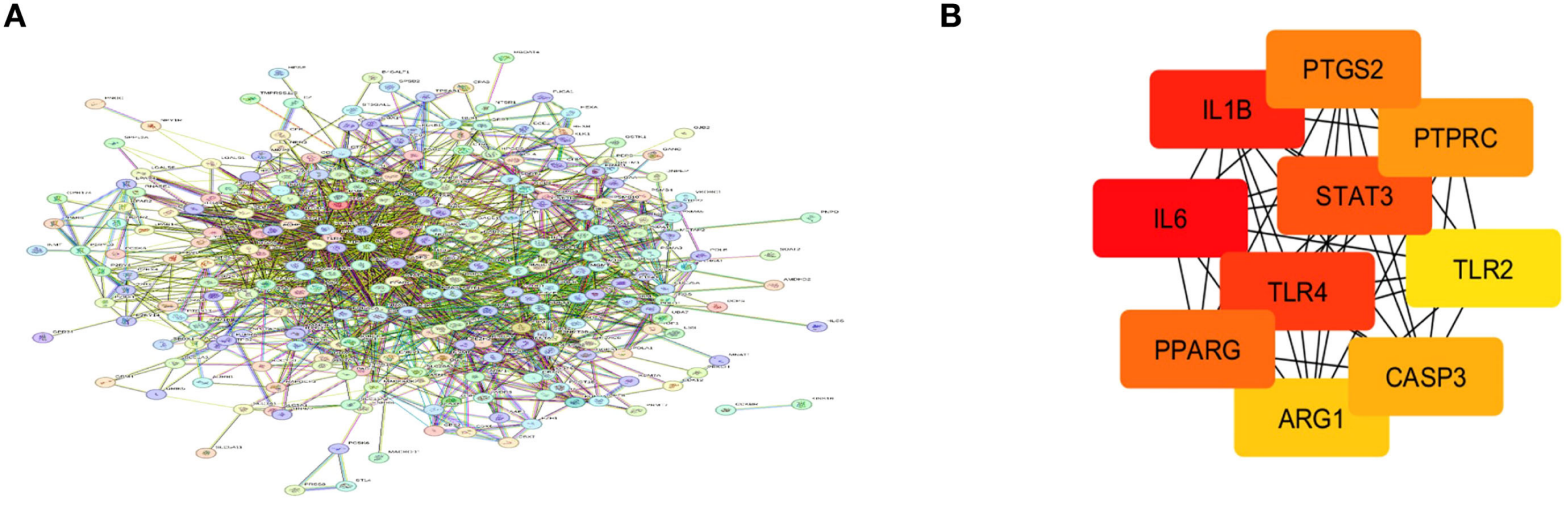
**(A)** Protein–protein interaction (PPI) network constructed using the STRING database for genes associated with otitis media. **(B)** Top 10 hub genes identified via the CytoHubba plugin in Cytoscape based on degree centrality. Node colors indicate ranking: red for highest connectivity, orange for intermediate, and yellow for lowest among the top 10.

STRING analysis was used to explore the functional protein–protein interactions (PPI) among the identified target genes as shown in **Figure 3A**. The analysis was conducted using the STRING database (v11.5), selecting the full STRING network option to include both physical and functional associations. A high confidence interaction score threshold of ≥0.7 was applied to ensure reliability. The interaction sources included a combination of evidence types such as experimental data, curated databases, text mining, and co-expression. The resulting PPI network consisted of 265 nodes and 1553 edges, with a statistically significant PPI enrichment p-value < 1.0e−16, indicating that the proteins are at least partially biologically connected. The network was imported into Cytoscape (v3.9.1) for further topological analysis. The top 10 hub genes were identified based on degree centrality using the CytoHubba plugin, revealing PTGS2, PTPRC, STAT3, IL1B, IL6, TLR4, TLR2, PPARG, ARG1, and CASP3 as key regulators within the network (**Figure 3B**).

### 3.6 Functional enrichment analysis

**Figure 4:**
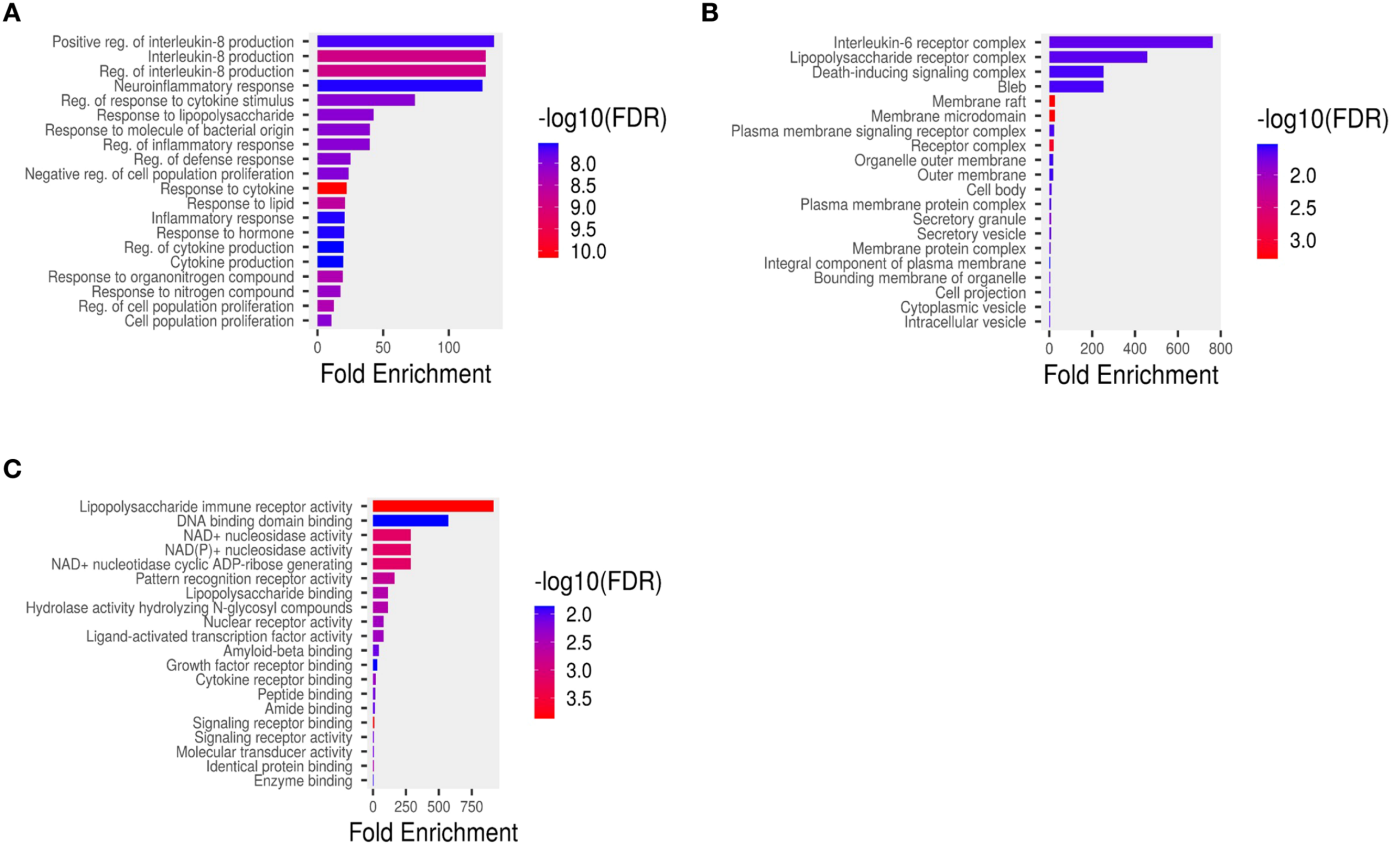
Functional enrichment analysis of hub genes based on Gene Ontology (GO) and pathway enrichment: **(A)** Cellular components, **(B)** Molecular functions, **(C)** Biological processes. The x-axis indicates the gene ratio, circle size reflects gene count, and color scale (red to blue) represents increasing false discovery rate (FDR).

Pathway enrichment analysis of hub genes was categorized into three Gene Ontology functional groups: Molecular Function (MF), Cellular Component (CC), and Biological Process (BP). GO analysis results represent the top 10 enriched terms of hub genes sorted by P-value. In the biological process category, the hub genes were associated with interleukin production, inflammatory response, regulation of proliferation, and regulation of cytokine production (**Figure 4A**). For cell component analysis, the hub genes were mainly present in interleukin-6 receptor complex, liposaccharide receptor complex, death inducing signalling complex and bleb (**Figure 4B**). The predicted molecular functions include lipopolysaccharide immune receptor activity, DNA binding domain activity, NAD(P)+ nucleosidase activity and NAD+ nucleosidase activity (**Figure 4C**).

### 3.7 Physical interaction and co-expression analysis using GeneMANIA

**Figure 5:**
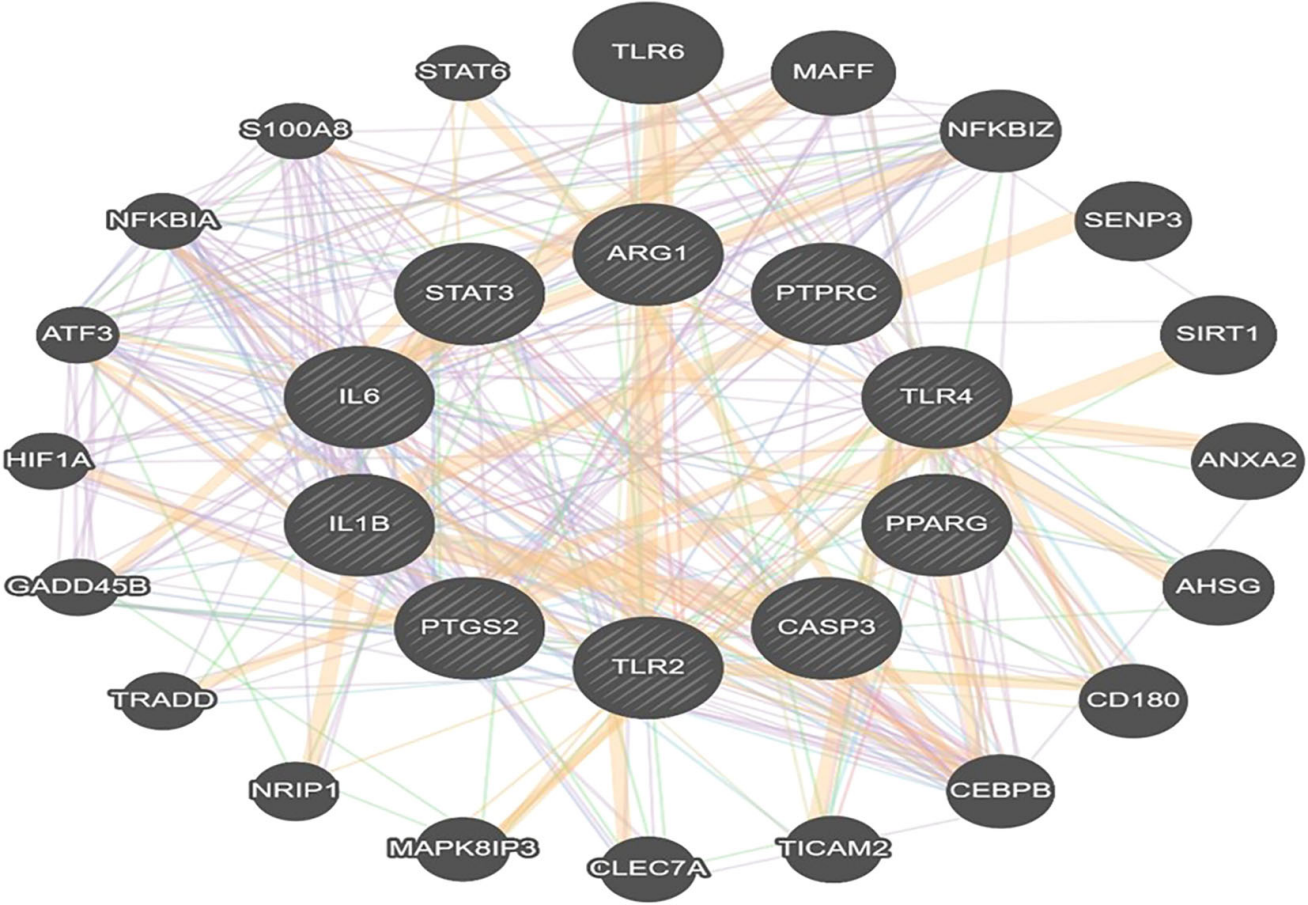
Gene–gene interaction network of hub genes generated using GeneMANIA. The network displays various types of interactions including physical binding, co-expression, prediction, co-localization, shared pathways, genetic interactions, and common protein domains.

GeneMANIA software was used to study the physical interaction and co-expression analysis of hub target genes to explore their functional associations. The identified hub targets were input to GeneMANIA, an online bioinformatics tool to investigate physical interaction and co-expression relationships. The analysis revealed that the hub targets accounted for 2.02% of physical interactions, 10.33% of co-localization, 2.96% of pathway analysis, 66.95% of co-expression,1.46% of shared protein domains and 16.22% of predicted interactions in otitis media (**Figure 5**).

### 3.8 miRNA target prediction and Integrated miRNA-target network construction

**Figure 6:**
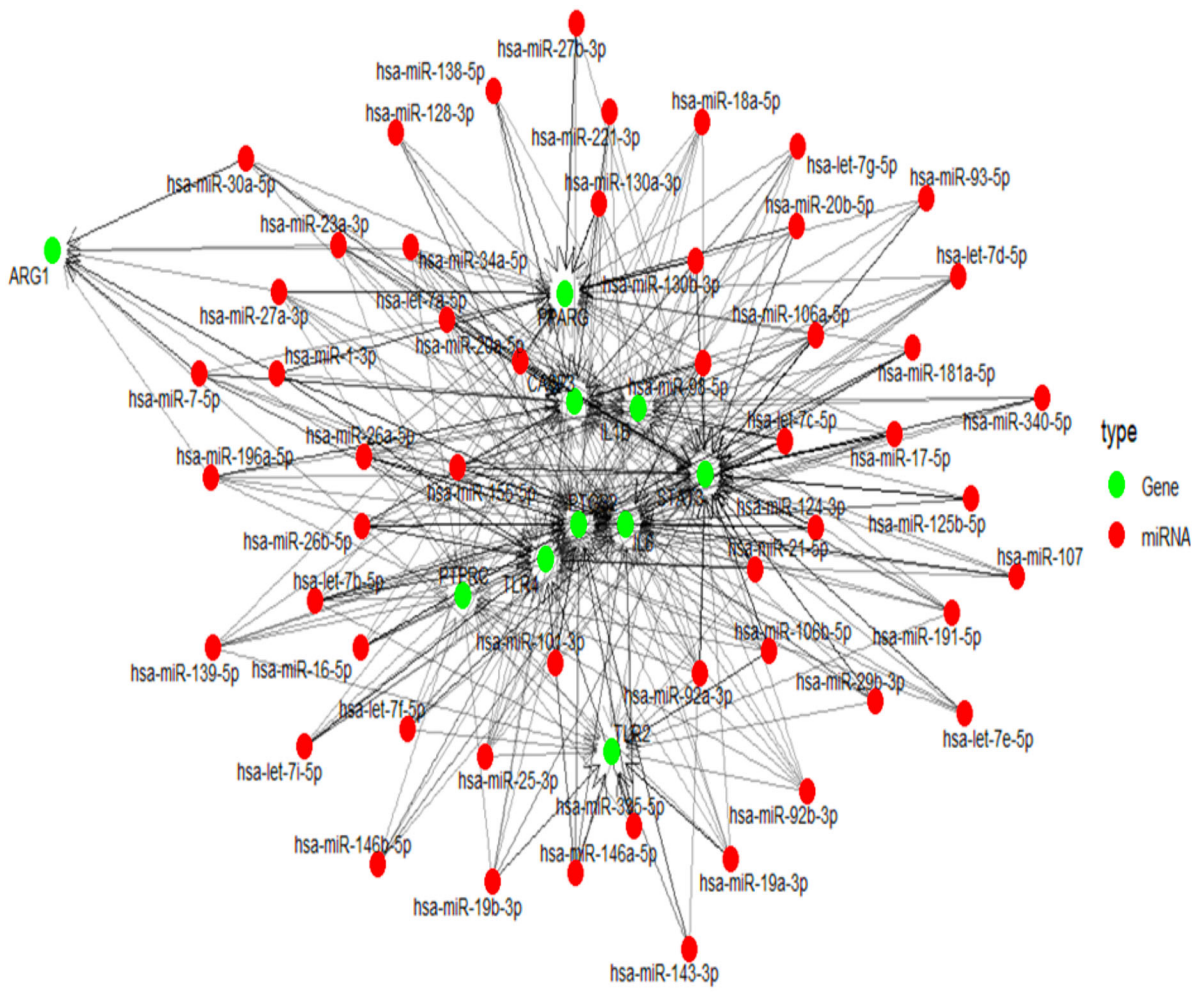
mRNA–miRNA interaction network, showing regulatory relationships. Red circular nodes represent miRNAs, while green nodes indicate their target mRNAs.

The multiMiR package of R was used to predict target miRNAs of hub genes from multiple databases, including miRTarBase, TargetScan, and miRDB (**Supplementary Table 4**). The miRNA–gene interaction network was constructed and visualized in R using the packages igraph for network topology, and ggraph for visualization using the Kamada-Kawai layout. The network consists of 474 unique miRNAs connected to 11 target genes through 1,452 regulatory interactions, which is depicted in **Figure 6**.

### 3.9 KEGG pathway analysis of miRNAs

**Table 1** shows the results of KEGG enrichment analysis to know the biological functions of identified miRNAs using DIANA-miRPath v3.0. The analysis revealed several significantly enriched pathways (p < 0.05), notably including the TGF-beta, MAP Kinase, TNF, Hippo, B cell and T cell receptor and Ras signaling pathways.

**Table 1:** The table presents the top signaling pathways significantly enriched by miRNA targets.

KEGG pathway	P-value	#Genes	#miRNAs
TGF-beta signaling pathway	4.13E-05	14	4
MAPK signaling pathway	0.000164	47	4
Neurotrophin signaling pathway	0.003509	24	4
Glycosphingolipid biosynthesis - lacto and neolacto series	0.004587	3	3
Ras signaling pathway	0.004587	33	4
Arrhythmogenic right ventricular cardiomyopathy (ARVC)	0.006333	13	3
Hepatitis B	0.009435	19	4
Hippo signaling pathway	0.013698	20	4
Prolactin signaling pathway	0.013698	15	4
N-Glycan biosynthesis	0.017151	7	4
TNF signaling pathway	0.020782	21	4
Signaling pathways regulating pluripotency of stem cells	0.023603	21	4
B cell receptor signaling pathway	0.037884	14	4
Pancreatic cancer	0.037884	12	4
T cell receptor signaling pathway	0.038953	19	4
Chagas disease (American trypanosomiasis)	0.040812	18	4

The table includes the pathway name, p-value, targeted gene number, and total number of contributing miRNAs.

### 3.10 miRNA -disease association network prediction

**Figure 7:**
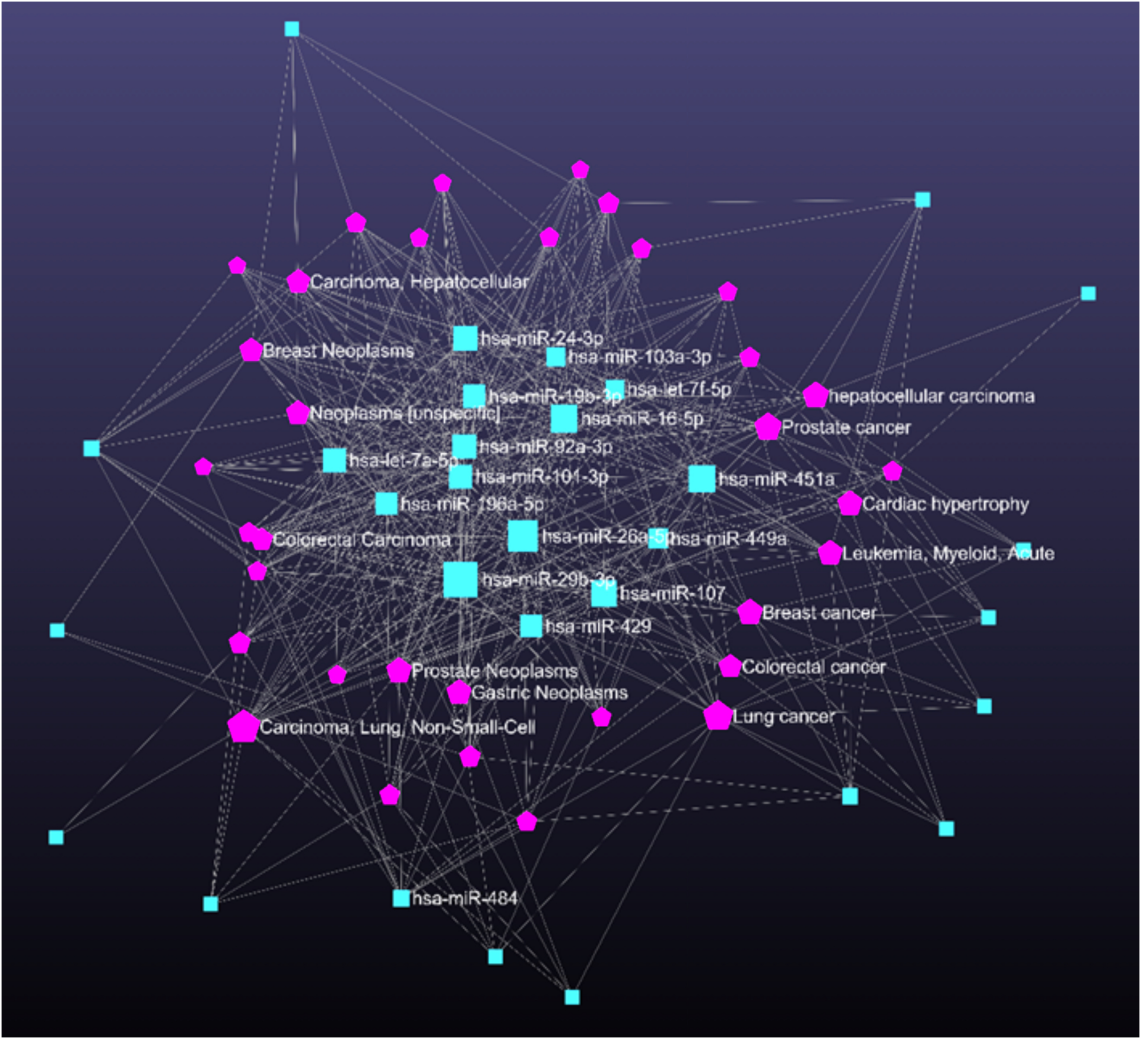
Network illustrating disease associations linked to key pathways regulated by disease-related miRNAs. Pink pentagons denote miRNAs and blue rectangles represent associated diseases.

Predicting miRNA-disease associations helped us to identify genes that can modulate biological processes linked to various diseases (**Figure 7**). The disease association degree was calculated for each miRNA with nodes and edges. The disease enrichment analysis using miRNet revealed strong associations between the identified miRNAs and several disease conditions, predominantly cancer-related pathologies. Notably, a significant number of the enriched miRNAs were linked to non-small-cell lung carcinoma, gastric neoplasms, prostate neoplasms, and colorectal carcinoma.

## 4 Discussion

In early childhood, acute otitis media frequently develops as a side effect of infections occurring in the upper respiratory tract. It is distinguished by effusion and the sudden onset of inflammation-related symptoms in the middle ear (Dermawan et al., 2025). The widespread usage of antibiotic treatment and prophylaxis of AOM has led to increased development of antibiotic resistance as well as disruptions of normal nasopharyngeal and gut microbiota (Florjan et al., 2024). This dysbiosis may allow opportunistic pathogens to proliferate and also impair the host immune homeostasis, rendering the patients most susceptible to infections. Recent research proposes that probiotic based treatment can stand as a promising alternative to antibiotics, owing to their ability to modulate the immune system and restore microbial balance. While research on the modulation of the gut microbiome as a therapeutic strategy for auditory disorders, including the gut–brain axis has been explored, and experimental studies have demonstrated the beneficial effects of probiotics in otitis media, our study takes a distinct systems biology approach (Kaytez et al., 2020). Specifically, we uniquely integrate miRNA -microbiota-host gene interaction networks, providing a comprehensive systems-level view of the gut–ear axis. To our knowledge, this is among the first attempts to apply network pharmacology and bioinformatics to investigate microbial and host crosstalk in otitis media. This integrative strategy reveals novel regulatory nodes and therapeutic targets that may not be apparent through traditional, single-omics or experimental approaches.

The gut–ear axis is an emerging concept suggesting that the gut microbiome may influence ear health through immune, metabolic, and neural pathways. While the gut–brain and gut–lung axes are well established, recent evidence indicates that gut microbial metabolites and immune signaling may affect the auditory system, particularly in inflammatory conditions like otitis media. The ear has its own microbiome, which may interact with gut microbes via systemic immune responses. Additionally, shared anatomical and physiological links, such as the Eustachian tube and vagal innervation, provide plausible routes for gut–ear communication. Further research is needed to clarify these interactions and their therapeutic potential (Graham et al., 2023).

Several gut microbiome strains are known to produce a wide range of metabolites. In this study, we utilised the MiMeDB database, with a focus on those produced by common probiotics such as Lactobacillus, *Bifidobacterium*, and *Streptococcus* species. These species are among the most commonly used probiotics and have well-documented evidence demonstrating their gut microbiota–modulating, anti-inflammatory, and immune-regulatory properties (Sarita et al., 2025; Sudha et al., 2022). This resulted in several metabolites, including acetate, butyrate, etc. SCFAs are important in maintaining various physiological processes. They provide energy for intestinal cells, gut barrier integrity improvement and also can stimulate anti-inflammatory signalling pathways. Additionally, metabolites, such as butyrate have gained importance due to their ability to influence gene expression. Butyrate treatment has been shown to influence key pathways such as cell cycle regulation, cellular differentiation, and fatty acid metabolism (Hodgkinson et al., 2023). These molecular effects position probiotic derived metabolites as promising therapeutic agents in inflammatory and metabolic diseases, including infections like Otitis Media, where modulation of host immune and epithelial responses is crucial. To connect these metabolites with otitis media pathology, we utilised the GSE dataset to identify differentially expressed genes. An intersection of these genes with the probiotic metabolite targets revealed 268 overlapping genes, which indicates that these genes may mediate the effects of gut metabolites on middle ear inflammation. In the PPI networks PTGS2, PTPRC, STAT3, IL1B, IL6, TLR4, TLR2, PPARG, ARG1, CASP3 were defined. PTGS2, which encodes COX-2, which releases prostaglandins, is expressed during inflammation and injury within the middle ear (Oka et al., 2024). *PTPRC* encodes the CD45 antigen, a protein which is essential for immune activation, particularly T/B cell receptor signalling (Li et al., 2023). Whereas STAT3 is involved in regulating tissue maintenance, its remodelling, and immune response, and has a role in various cancer progression (Xia et al., 2023). Pro-inflammatory cytokines like IL-1β and IL-6 have a significant involvement in otitis media development and its progression. They contribute to the inflammatory response by recruiting immune cells, inducing tissue damage and stimulating the release of additional pro-inflammatory factors (Serban et al., 2021). Research has proved that probiotics show immunomodulatory effects by modulating the release of cytokines, particularly interleukins (ILs), which play an important role in both adaptive and innate immune response (Azad et al., 2018). This suggests that probiotics derived metabolites support immunological homeostasis and may help treat inflammation-related disorders like otitis media by altering cytokine profiles, such as by increasing anti-inflammatory mediators and inhibiting pro-inflammatory cytokines. Otitis media can arise as a consequence of infection in the upper respiratory tract and dysfunction of the Eustachian tube. If the pathogens causing infections are not recognised early or if the immunity is compromised, it can lead to chronic infections. Early recognition of pathogens and initiation of immune responses are critically mediated by Toll-like receptors, including TLR4 and TLR2. Altered TLR expression, whether delayed, absent, or excessive, is linked with the onset, persistence, and progression of otitis media, highlighting its importance in the disease’s pathogenesis (Jung et al., 2021). It is also proven that gut microbial components and metabolites interact with these TLRs, affecting immune and metabolic homeostasis. These interactions help the body distinguish between beneficial and harmful microbes, maintaining a balanced immune response (Chen et al., 2024). In chronic otitis media, CASP3 is over-expressed and enzymatically activated, reflecting a persistent but insufficient attempt at apoptotic clearance in the face of ongoing inflammatory and proliferative stimuli. This dysregulated balance between cell death and regeneration helps drive the pathological mucosal hyperplasia and tissue remodelling seen in chronic middle-ear disease (Leichtle et al., 2022).

Functional enrichment analysis of hub genes gained insight onto their underlying mechanism in otitis media. The Gene Ontology (GO) categorization highlighted that identified genes are predominantly involved in immune and inflammatory regulation, like interleukin production, cytokine regulation, inflammatory response, and cell proliferation. These are critical to the pathophysiology of otitis media particularly in its progression from acute to chronic stages. Cellular component analysis showed that genes are found to be localised in key immune-related complexes such as those involved in IL-6 signalling, bacterial component recognition, and apoptosis initiation, underscoring their role in pathogen recognition and inflammatory signalling. Furthermore, the Molecular Function (MF) terms identified functions which are closely linked to microbial sensing and downstream immune activation, including lipopolysaccharide receptor activity, DNA-binding, and NAD+ nucleosidase activity.

MicroRNAs(miRNAs) are known to modulate gene expression, influencing the development and recurrence of both acute and chronic inflammatory conditions in the middle ear. miRNAs can regulate multiple mRNA targets, while a single mRNA can be regulated by several miRNAs (Kotowski et al., 2023). To understand this complex, we developed a gene-miRNA interaction network. The high number of interactions relative to the small number of target genes indicates intensive miRNA regulation where multiple miRNAs potentially converge on shared gene targets. Such a pattern suggests that these target genes may play critical regulatory roles in otitis media, possibly acting as signalling hubs or effectors in inflammation and immune pathways. Genes such as IL6, STAT3 and TLR4 were highly targeted. This strengthens their known involvement in innate immunity and inflammatory responses. Several other studies have focused on miRNAs like miR-210 and miR-146a, which were also identified through our study. These are implicated in the inflammatory response and immune cell behaviour in otitis media. miR-210 plays a significant role in hypoxia-related processes, and its expression is altered in otitis media. Similarly, miR-146a is known to regulate the inflammatory response and its dysregulation is associated with otitis media (Samuels et al., 2016; Zhang et al., 2020).

KEGG pathway analysis indicates enrichment of miRNAs in immune modulation, inflammation, epithelial cell homeostasis, and cell proliferation related pathways. These are the mechanisms that are central to the pathogenesis of otitis media. Notably, the TGF-beta, which ranked among the top enriched pathways (p = 4.13E-05), play crucial roles in regulating immune responses and mucosal repair, both essential for controlling middle ear inflammation and facilitating recovery (Yamamoto-Fukuda et al., 2023).

A significant number of the enriched miRNAs were linked to non-small-cell lung carcinoma, gastric neoplasms, prostate neoplasms, and colorectal carcinoma. Interestingly, beyond cancer associations, conditions such as heart failure and acute myeloid leukaemia were also enriched, suggesting that the deregulated miRNAs might have systemic roles in regulating inflammation and immune responses. Given the overlapping pathways between chronic inflammation in cancer and otitis media, these findings provide a potential mechanistic link and support the therapeutic relevance of the identified miRNAs in inflammatory diseases like otitis media.

## 5 Conclusion

Our study aimed to investigate the therapeutic role of gut probiotic derived metabolites in managing otitis media through a bioinformatics approach. Our result demonstrated a strong link between metabolites, their target genes and miRNA-mediated regulatory mechanisms in otitis media. The identified genes and metabolites are found to be involved in inflammatory and immune regulatory pathways, which are central to otitis media pathogenesis. The miRNAs were found to be involved in pathways including TGF-beta and MAPK, which highlights their pivotal role in regulating immune responses, inflammation, and tissue repair mechanisms associated with otitis media. This suggests that certain miRNAs can modulate these pathways and so can be considered as potential targets for developing new therapeutic interventions aimed at modulating immune response and promoting mucosal healing. Targeting these molecular players could pave the way for the development of novel miRNA-based therapeutics or microbiota-targeted strategies such as probiotic formulations or metabolite modulators. Overall, our study highlights the potential of the gut–ear axis as a valuable target for microbiome-based therapeutic strategies, particularly by utilizing probiotic metabolites to modulate gene expression and inflammation in otitis media. A key limitation of this work was the limited availability of published literature directly addressing the role of gut probiotics in otitis media, suggesting the need for further experimental and clinical studies in this emerging area. Future studies are needed to experimentally validate these interactions through both *in vitro* and *in vivo* assays, and to assess their clinical relevance using patient-derived samples or biomarker-based cohort studies.

## Data Availability

The original contributions presented in the study are included in the article/**Supplementary Materials** further inquiries can be directed to the corresponding author/s.
